# The Hepatoprotective Effect of Haoqin Qingdan Decoction against Liver Injury Induced by a Chemotherapeutic Drug Cyclophosphamide

**DOI:** 10.1155/2015/978219

**Published:** 2015-05-26

**Authors:** Xiaojiang Li, Baole Li, Yingjie Jia

**Affiliations:** Department of Oncology, First Teaching Hospital of Tianjin University of Traditional Chinese Medicine, 314 An Shan Xi Dao, Nan Kai District, Tianjin 300193, China

## Abstract

Haoqin Qingdan decoction (HQQD), a modern Chinese formula, has been widely used in Eastern Asia. Our study focuses on the hepatoprotective effect of HQQD against cyclophosphamide-induced hepatotoxicity. S180, a kind of ascites tumor cells, was used to establish S180-bearing mice, followed by the injection of cyclophosphamide (CP, 80 mg/kg) every other day for 5 times. HQQD was used intragastrically at the dose of 80 g/kg, 40 g/kg, and 20 g/kg twice a day for 12 days. HL-7702 hepatic cell line was incubated with HQQD-medicated serum. Then we detected the effects of HQQD on (i) tumor suppression; (ii) morphological examination; (iii) SOD, MDA, GSH, ALT, and AST; (iv) cleaved caspase-3 expression and (v) cellular viability. CP caused dramatic elevations of AST, ALT, and MDA, while HQQD notably attenuated these elevations. SOD and GSH were notably increased, which were efficiently attenuated by HQQD. CP injection significantly increased apoptosis by increasing cleaved caspase-3 expression, which was obviously inhibited by HQQD, accompanied by the improvement of cells viability. Histopathological examinations supported the above findings. Therefore, HQQD may protect liver tissue through attenuating oxidative stress and the caspase-3-dependent intrinsic apoptosis induced by CP, which suggests the potentially therapeutic effect of HQQD in the use of alkylating agent for cancer chemotherapy.

## 1. Introduction

Currently, cancer is the common disease which seriously endangers human health and lifespan, with its high morbidity and mortality. Chemotherapy is by far one of the primary methods employed in cancer treatment, the efficiency of which is generally elevated by using a combination of different types of antineoplastic drugs [1]. Nevertheless, chemotherapy can produce different degrees of damage to the normal tissues because of its low selectivity to normal tissues and cancer tissues. Cyclophosphamide (CP) is an alkylating agent widely used in cancer chemotherapy [2]. The injury of normal tissues is the major limitation of using CP, which gives rise to numerous side effects [3]. Therapeutic dose of CP causes liver disorders [4], nephrotoxicity [5], immunotoxicity, and mutagenicity [6] which lead to gonadal toxicity, as a side effect of the drug. The liver is the center of drug metabolism and bioconversion, rich in a variety of biotransformation enzymes. Drug bioconversion in the liver usually generates inactive metabolic end products which are excreted from the body but can also generate toxic metabolites [7]. Consequently, the mitigation of the liver damage caused by CP and strengthening of drug efficacy are vital clinical problems to be solved. Further therapeutic agent development, especially the pharmacodynamic constituents from natural herbs in complementary and alternative medicine (CAM), has been regarded as the focus in the combat with CP-induced liver damage.

Among CAMs, the utility of traditional Chinese medicine is related to the concepts that it can alleviate toxic effects associated with chemical treatments and improve quality of life [8, 9]. For example, multiple ingredients from Chinese herbs counteract chemotherapy-induced gastrointestinal toxicity and kansui-induced hepatocyte cytotoxicity [10, 11]. Haoqin Qingdan decoction (HQQD), a traditional Chinese formula widely used for the treatment of different diseases [12, 13], consists of herbal materials of* Artemisinin*, bamboo shavings,* Pinellia ternata*,* Indian buead*,* Scutellaria baicalensis*,* Aurantii fructus*,* Aurantii nobilis pericarpium*, and Biyu powder. Although there are a lot of researches regarding HQQD, few of them are involved in the hepatotoxicity induced by CP. Hence, this study investigated the hepatoprotective potential of HQQD against liver injuries induced by CP in terms of antioxidation and antiapoptosis.

## 2. Materials and Methods

### 2.1. Animals

The experiment was approved by the Institutional Animal Care and Use Committee of Tianjin University of Traditional Chinese Medicine and in accordance with the principles outlined in the NIH Guide for the Care and Use of Laboratory Animals. Kunming male mice (SPF grade), weighed 19.6 ± 2.3 g, were purchased from Department of Laboratory Animal Science, Peking University Health Science Center (Certificate number 0182799). All the mice were kept in standard cages at 25 ± 1°C under a 12 h light/dark cycle and fed a rodent standard diet with free access to water.

### 2.2. Tumor Inoculation and Cyclophosphamide Administration

S180 ascitic cells (5 × 10^6^ cells) were mixed with saline at ratio of 1 : 4 and transplanted subcutaneously into the right armpit region of the mice (0.2 mL/mice). The diameter of the tumor size was about 1 cm after the observation for 7 days. CP at the dose of 80 mg/kg was injected intraperitoneally, every other day for 5 times. Except for the untreated S180-bearing mice model which was used as control, all mice received CP administration.

### 2.3. Cell Culture

The human HL-7702 hepatic cell lines were cultured in T-25 tissue flasks at 5% CO_2_ and 37°C humidified atmosphere using DMEM culture media supplemented with 10% FBS. The hepatic cells were maintained via two to three passages each week.

### 2.4. HQQD Preparation and Administration

The composition of HQQD was* Artemisia apiacea* (12 g), bamboo shavings (18 g),* Pinellia ternata* (9 g),* Poria cocos* (18 g),* Scutellaria baicalensis* (18 g), raw* Fructus aurantii* (9 g), dried tangerine peel (18 g), and* Biyusan* (18 g). All of these herbal materials (Tianjin Chinese Herbal Medicine Co., Ltd., Tianjin, China) followed the standards described in the Pharmacopoeia of Chinese Medicine. Firstly, the mixture of the eight herbs was boiled in deionized water (Thermo Scientific MicroPure) for 40 min and filtrated with stainless steel filter mesh to acquire the herbal extract. The left dregs were boiled for the second time for 20 min and filtrated again. The filtration liquids of the two times were mixed and heated to evaporate at a concentration of 2 g/mL.

In the preliminary experiment, we observed the effect of individual use of HQQD on S180-bearing mice. As the results shown in Figure S1 (in Supplementary Material available online at http://dx.doi.org/10.1155/2015/978219), low, moderate, or high dose of HQQD alone could significantly reduce the tumor weight as compared with the control mice which suggested an effectiveness of HQQD. Meanwhile, CP suppressed the thymus and spleen index which were reversed by individual use of HQQD. These results indicated a protective effect of HQQD alone on CP injury. Considering the effectiveness of HQQD individual use, we conferred that HQQD might reduce CP injury. Therefore, based on the CP treatment, the mice received the HQQD administration additionally. Mice in moderate-dosage HQQD treatment group were administrated with 40 g/kg decoction twice a day for 12 days and the HQQD treatment was started at the beginning of CP administration, which was calculated according to the daily human clinical dosage (Food and Drug Administration, 2002). The animals in high-dosage and low-dosage groups received HQQD in the same manner, but at 2-fold of moderate dose and half the moderate dose, respectively (80 g/kg, 20 g/kg).

### 2.5. HQQD Medicated Serum Preparation

One hour and two hours after the last HQQD administration, totally 8 mL blood in each group was drawn (0.5–0.8 mL/mouse) from eyeball and centrifuged at 3000 ×g for 15 min to acquire the serum. The serum from S180-bearing mice administrated with saline was used as the baseline and signed as MS_base_ (mice serum, MS). The HQQD medicated serum from two time points was mixed and incubated at 56°C for 30 min for complement system inactivation. The serum from four groups was signed as MS_base_, MS_low_, MS_mod_, and MS_high_, respectively. The normal cultured hepatic cells without any treatment were used as control. The HQQD medicated serum volume was 20% and the incubated time was 24 hours.

### 2.6. Evaluation of Tumor Growth

Twenty-four hours after the last administration, S180 sarcoma tissues in different treated groups were stripped out and weighed. Tumor inhibition rate was calculated as follows: tumor inhibition rate = (1 − average weight of tumors in treatment group/average weight of tumors in control group) × 100%.

### 2.7. Morphological Examination

The histopathological evaluations of hematoxylin-eosin staining (H&E) staining in each group were conducted by standard histological techniques. The hepatic tissue was fixed in 10% buffered formalin and embedded in paraffin. The 5 *μ*m sections were stained with H&E.

### 2.8. Detection of SOD, MDA, and GSH

The hepatic tissues were moved out and rinsed with PBS and dried with filter paper, followed by being weighed accurately with balance (sensitivity: 1/10000). The tissues were rapidly dissected and homogenized in nine volumes of ice-cold phosphate buffered saline (PBS). The lysis solution was centrifuged at 3,000 rpm for 20 min and the proteins in the supernatants were quantified with coomassie brilliant blue (Bradford method). The SOD activity was estimated using xanthine oxidase method. The procedure was conducted with SOD commercial kit and the result was obtained by the microreader (Tacan, Swaziland) with the absorbance of 550 nm. The xanthine/xanthine oxidase (XO) assay was used to estimate superoxide dismutase (SOD) activity (U/mg protein) by measuring the amount of reduced nitro blue tetrazolium (NBT), with one unit of SOD defined as the amount of protein that inhibits the rate of NBT reduction by 50% [14]. The level of lipid peroxidation was expressed as the content of malondialdehyde (MDA) which was examined by thiobarbituric acid method (TBA). The level of lipid peroxidation was expressed as the content of malondialdehyde (MDA) which was examined by thiobarbituric acid method (TBA). The procedure was conducted with MDA commercial kits according to the manufacturer's instruction (Beyotime Biotechnology, Jiangsu, China), with the test wavelength of 532 nm [15]. GSH activity was detected by precipitating tissue homogenates with 15% sulfosalicylic acid and processing the supernatant by the oxidized glutathione recycling procedure and was tested at the absorbance of 412 nm [16].

### 2.9. Serum Biochemical Analysis

Whole-blood samples obtained from the abdominal aorta were centrifuged for 15 minutes at 3,000 ×g to obtain the serum. Serum samples were stored at −70°C until biochemical analyses within 5 days. The serum levels of aspartate transaminase (AST), alanine transaminase (ALT), and alkaline phosphatase (ALP) were determined using an Auto Chemistry Analyzer (AU400; Olympus, Tokyo, Japan).

### 2.10. Cell Viability

HL-7702 hepatic cells at 1 × 10^4^ cells/mL were seeded on 96-well plates. A series of CP concentration (5, 2.5, 1.25, 0.625, 0.3125, 0.1563, 0.0781, 0.0391, 0.0195, 0.0098, 0.0049, and 0.0024 mg/mL) was used to determine the IC50 and the final concentration of CP was 1.2 mg/mL in the culture medium. The serum addition volume was 20%, accompanied by the control of normal cultured cells with blank mice serum and the normal cultured cell with FBS. After incubation for 68 h, fluids in 96-well culture plates were changed to DMEM to avoid background interference and 10 *μ*L of Cell Counting Kit (CCK-8) was added in each well to incubate for 2 hours, followed by measurement using a microplate reader with a test wavelength of 450 nm with 620 nm as reference wavelength.

### 2.11. Apoptosis Detection by Western Blot

At 24 hours after incubation, HL-7702 hepatic cells were washed with PBS and scraped in RIPA lysis buffer (50 mM Tris and 150 mM NaCl, pH 7.4, containing 1% Triton X-100, 1% Nonidet P-40, 0.5% sodium deoxycholate, 0.1% sodium dodecyl sulfate, 1 mM phenylmethylsulfonyl fluoride, 15 *μ*g/mL leupeptin, 71 *μ*g/mL phenanthroline, and 20 U/mL aprotinin). The insoluble material was removed by centrifugation at 12,000 ×g for 20 min. Twenty micrograms of proteins was processed by SDS-PAGE separation in 12.5% gel and transferred to a 0.45 *μ*m polyvinylidene fluoride (PVDF) membrane. The PVDF membrane was then incubated with TBS (40 mM Tris, pH 7.6 and 300 mM NaCl) containing 5% nonfat dry milk for 1 h at 37°C to block nonspecific binding sites. The PVDF membrane was incubated with primary antibodies (1 : 1000 dilution) against cleaved caspase-3 or against *β*-actin as controls, followed by incubation with the corresponding HRP-conjugated secondary antibodies (1 : 10,000 dilution). Immunoreactive proteins were detected by enhanced chemiluminescence according to the instructions of the manufacturer (Pierce, Rockford, IL). Three animals were used in each experimental group.

### 2.12. Statistical Analysis

The experimental data are represented as mean ± standard deviation (SD). One-way analysis of variance (ANOVA) was used to determine statistically significant differences. Also, post hoc Tukey's honest significant difference (HSD) tests were performed at the second stage for multiple comparison. A *p* value of <0.05 was considered to be statistically significant.

## 3. Results

### 3.1. HQQD Treatment Inhibits Tumor Growth in S180-Bearing Mice

**Figure 1:**
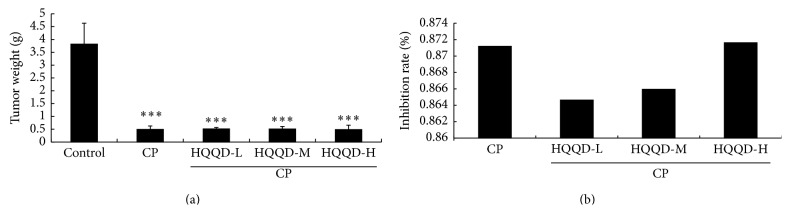
HQQD inhibits the tumor growth. (a) The tumor weights treated by HQQD with low (20 g/kg), moderate (40 g/kg), and high dosage (80 g/kg). (b) The inhibition rate (%) produced by HQQD. Tumor inhibition rate calculated as percent compared to control. Data displayed as mean of 10 mice in each group. The experiment was independently repeated for 3 times. ^*∗∗∗*^ *p* < 0.001 versus control group.

To measure tumor growth inhibition caused by HQQD treatment, tumor tissues were measured and weighed.  Figure 1(a) shows that both CP treatment and CP plus HQQD treatment inhibited the tumor growth compared with control group (*p* < 0.001). HQQD-H combined with CP was more effective in reducing the tumor growth compared with HQQD-M and HQQD-L, or CP alone. As shown in Figure 1(b), CP inhibition rate was 87.11 percent compared with control group; the inhibition rate for CP combined with HQQD-H was 87.16 percent. From the above results, we found that HQQD combined with CP could inhibit the tumor growth, with the high dose of HQQD having the most obvious inhibitory effect.

### 3.2. HQQD Reverses Liver Damage Induced by CP from the Pathological Changes in H&E Slides

**Figure 2:**
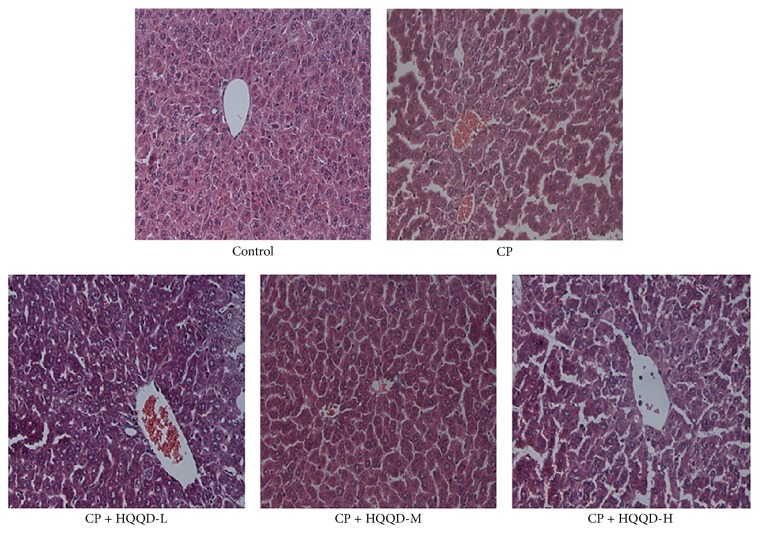
Representative photomicrographs of H&E staining on hepatic morphology show that HQQD reverse liver injury induced by CP from the pathological feature. All of the mice were killed at the end of experiment, and liver tissues were quickly harvested for histopathology analysis. The structure in the mice treated by HQQD was recovered to some extent, with the less necrosis, neutrophils infiltration, and cellular edema.

As shown in Figure 2, through H&E light micrographs for histopathological study, normal control group mice showed normal architecture, characterized by complete and clear liver tissue structure, close-connected liver cells, and normal hepatic lobule. In the CP-treated liver tissue, many hepatic structure disorders as well as infiltration of acute inflammatory cells were observed. In addition, there also existed liver cell edema, vacuolar degeneration in the perivascular tissue, focal necrosis, and a large number of inflammatory cells infiltration. The structure in the mice treated by HQQD was recovered to some extent. Under microscope, the necrosis was reduced to a less degree, with a less neutrophils infiltration in the hepatic portal area and less cellular edema.

### 3.3. HQQD Regulates SOD, MDA, and GSH Activities in Hepatic Tissues

**Figure 3:**
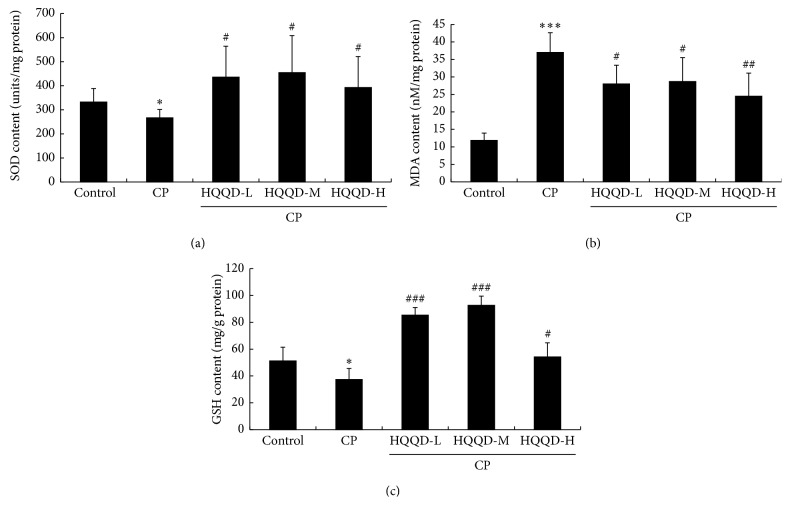
HQQD effects on oxidative stress parameters: (a) changes in SOD activity in different treated mice; (b) changes in MDA concentration in different treated mice; (c) changes in the GSH activity in different treated mice. Data expressed as mean ± SD for 7 mice in each group. ^*∗*^ *p* < 0.05 versus control group, ^*∗∗∗*^ *p* < 0.001 versus control group; ^#^ *p* < 0.05 versus the CP group, ^##^ *p* < 0.01 versus the CP group, and ^###^ *p* < 0.001 versus the CP group.

The activities of hepatic antioxidant enzymes SOD showed a significant decrease in CP-induced mice (Figure 3(a)). Along concurrent administration with CP, HQQD of low, moderate, and high dose significantly increased the activities of SOD (*p* < 0.05). Liver lipid peroxidation (LPO), estimated as MDA, showed a significant (*p* < 0.001) increase in CP-induced mice when compared to the control group. Supplementation of 80 g/kg, 40 g/kg, or 20 g/kg HQQD significantly ameliorated the increased MDA levels (*p* < 0.05, *p* < 0.01, Figure 3(b)), with the high dose of HQQD having the most obvious effect. Similarly, CP administration produced a significant (*p* < 0.05) decrease in GSH content in the hepatic tissue of CP-injured mice. Per contra, HQQD coadministration significantly increased hepatic GSH content when compared to mice that received CP only as depicted in Figure 3(c).

### 3.4. HQQD Inhibits ALT and AST Levels in Hepatic Tissues

**Figure 4:**
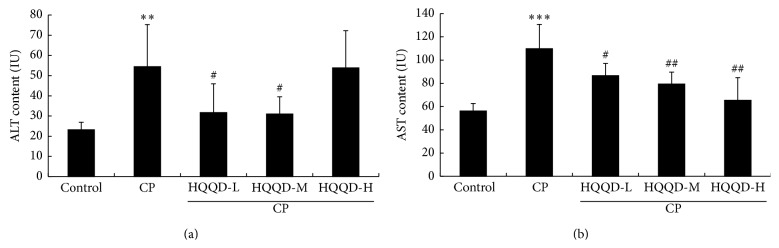
Suppressive effect of HQQD on elevated serum enzyme activities. HQQD with low and moderate dose significantly inhibited the ALT (a) and AST (b) levels. HQQD with high dose significantly inhibited AST level. Data expressed as mean ± SD for 7 mice in each group. ^*∗∗*^ *p* < 0.01 versus control group, ^*∗∗∗*^ *p* < 0.001 versus control group; ^#^ *p* < 0.05 versus the CP group, ^##^ *p* < 0.01 versus the CP group.

With data describing the suppression of HQQD on serum liver marker enzymes, ALT and AST activities were represented in Figure 4. CP-induced mice displayed a significant (*p* < 0.001, *p* < 0.001) increase in sera ALT and AST activities when compared to control mice. Concurrent administration of HQQD-M or HQQD-L along with CP produced a potential (*p* < 0.05) alleviation of the altered serum levels of ALT and AST. The inhibitory effects of HQQD-H on ALT activities were not observed.

### 3.5. HQQD Reverses the Increase of Cleaved Caspase-3 Expression Level in Liver Tissues Induced by CP

**Figure 5:**
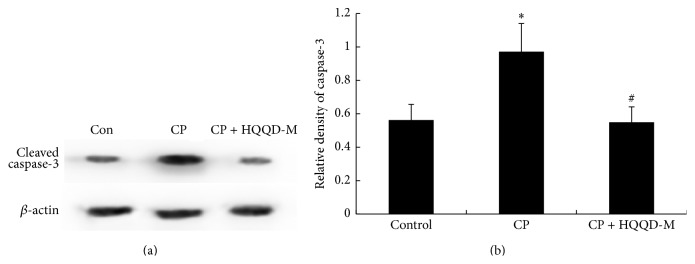
Effect of HQQD on cleaved caspase-3 expression. (a) The effect on cleaved caspase-3 protein formation caused by 80 mg/kg CP was examined at dose of 40 g/kg of HQQD. Data were normalized to the *β*-actin protein, which was used as an internal control. (b) Bar graphs were shown as means ± SD of three mice per group. Values represented the mean optical density ratio relative to the internal control. ^*∗*^ *p* < 0.05 versus the control group; ^#^ *p* < 0.05 versus the CP group.

To explore mechanisms of the protective effect that HQQD plays on liver injury induced by CP, we tested the protein level of cleaved caspase-3 in mice hepatic tissue. As shown in Figure 5, cleaved caspase-3 expression in hepatic tissue in CP group increased by 1.73-fold compared with control group (*p* < 0.05). After supplement of HQQD at 40 g/kg, cleaved caspase-3 level was reduced by 43.6% (*p* < 0.05) compared with CP group, indicating that HQQD was likely to alleviate liver injury induced by the chemotherapeutics through antiapoptosis.

### 3.6. HQQD Increases the Hepatic Cell Viability Induced by CP

**Figure 6:**
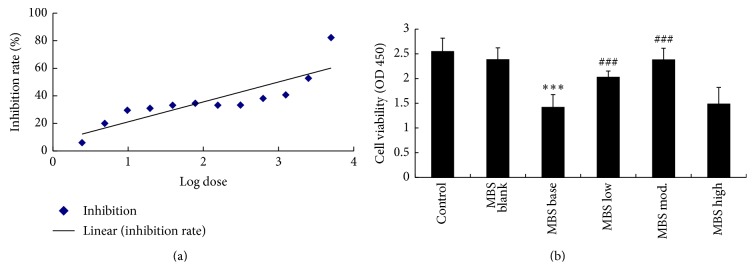
(a) Trendline represented the relation between cell viabilities and different dose of CP. (b) Bar graphs showed the changes of hepatic cells viability determined via CCK-8 assay. HQQD of moderate and low dose of medicated serum improved the cells viability. Data expressed as mean ± SD for 6 wells in each group. ^*∗∗∗*^ *p* < 0.001 versus the control group; ^###^ *p* < 0.001 versus the CP group.

Initially, we detected the IC_50_ of CP for the HL-7702 cell line and found that all the concentration of CP had inhibitory effect on hepatic cell viability (Figure 6(a)). After calculating the inhibition rate, linear formula was acquired as *y* = 0.1284*x* + 0.1078, and IC_50_ was 1.145 mg/mL when *Y* was defined as 50%. We used CP dose as 1.2 mg/mL in the following cell viability assay. The cell viability in the MS_blank_ showed no difference compared with the control, indicating that the mice serum had no effect on the basal state of cell. It was obvious that the cell viability treated by CP-medicated serum was decreased significantly compared with the control or MS_blank_ but recovered by MS_low_ and MS_mod_ to different level (Figure 6(a), *p* < 0.001).

## 4. Discussion

Chemotherapy constitutes an important component and is used in the therapy of various kinds of tumors. Cyclophosphamide (CP) is one kind of chemotherapeutics that are most commonly used, which can be used alone or in combination with other chemotherapeutics. Although CP has showed commendable clinical therapeutic effects, its hepatic toxicity cannot be ignored [7]. The antitumor mechanism of CP is primarily cross-linking guanine bases in DNA double-helix strands which results in the disruption of DNA function and cell death [17, 18]. Liver injury is one of the most common toxic effects of CP clinically [19]. Many CAMs show significant improvements in chemotherapy- or radiotherapy-related side effects and TCM is the most common means of CAMs in China. Haoqin Qingdan decoction (HQQD), as a traditional Chinese medicine, is usually used in China as a complementary treatment to Western treatments that are recommended for digestive system disease. Nevertheless, there were limited reports about the treatment of HQQD for liver injury induced by CP. Our present study provides the important evidence of the beneficial effects of HQQD on liver injury resulting from CP administration.

We firstly present the tumor suppression of HQQD in S180-bearing mice model induced by CP. In this study, we show that HQQD combined with chemotherapy drug CP inhibited tumor growth, especially for the high dose of HQQD which had a higher inhibition rate than CP treatment alone. Our results suggest that HQQD high dose treatment could improve the efficiency of chemotherapeutic drugs. CP is extensively metabolized by the liver cytochrome P450 system, which probably causes sinusoidal obstruction syndrome, resulting in a direct toxic effect on hepatic sinusoidal cells, thus inducing necrosis, obstruction, and obliteration of hepatic veins [20, 21]. Consistent with those previous studies, histological changes including liver congestion, disorganization of hepatic cords, and ballooning degeneration could be observed in H&E slides. Our data further suggests that following HQQD administration, morphological damage is significantly attenuated, which may be one of the main reasons of the hepatoprotective effect of HQQD.

Oxidative damage initiated by ROS can cause lipid peroxidation, resulting in altered membrane structure and enzyme inactivation. It is the action of abstraction of a hydrogen atom from the side chain of polyunsaturated fatty acids in the membrane [22]. Our present data revealed that CP administration produced a marked oxidative impact, as evidenced by the significant increase in MDA as well as decrease in SOD and GSH levels. The SOD constitutes dismutation of endogenous cytotoxic superoxide radicals to H_2_O_2_ and molecular oxygen that are deleterious to polyunsaturated fatty acids and proteins [23]. The reduction in the activities of the SOD and GSH and increase in MDA could reflect the adverse effects of CP, which caused an imbalance in the antioxidant system in the liver tissue. HQQD administration restored these enzyme levels near to normal by balancing the antioxidant defense system. Some active compounds may have originated from HQQD such as artemisinin and were reported to regulate GSH [24]. This might be ascribed to the free radical scavenging/antioxidant properties of the phytochemical constituents present in HQQD.

It was reported that a cohort of 85 Chinese breast cancer patients who received adjuvant chemotherapy with CP (600 mg/m^2^) every 3 weeks were found to have a higher incidence of hepatotoxicity as indicated by raised ALT activity [25]. Elevations in sera AST and ALT activities following the administration of CP were attenuated by concurrent treatment with low or moderate dose of HQQD. These changes in serum enzyme activities were consistent with the extent of histological and structural damage observed in liver tissue, which indicated the morphofunctional recovery of damaged liver by the administration of HQQD. On the other hand, HQQD given at dosages of 80 g/kg had no significant effect on changes in the activities of ALT after exposure to CP. These findings suggested that the concomitant use of an appropriate dose of HQQD, such as 40 g/kg and 20 g/kg, was capable of protecting against CP-induced hepatic toxicity.

Cell apoptosis is involved in the CP-induced acute hepatic toxicity [26, 27], characterized by activation of caspase-3, a key cell death protease [28]. CP-induced cleaved caspase-3 protein in the liver was significantly attenuated by concurrent treatment with HQQD, and the degree of suppression reached control levels. In addition, the hepatic cell viability was also improved by the HQQD low or moderate dose of medicated serum, which was considered as a reason for the antiapoptosis effect of HQQD. A future important strategy to reduce CP-induced hepatotoxicity will involve an exploration of other compounds in HQQD that have high inhibitory potency toward the stimulated caspase-3-mediated extrinsic pathway.

## 5. Conclusion

Overall, the present study demonstrated that HQQD has hepatoprotective effect which may be partly due to attenuating oxidative stress and the hepatic caspase-3-dependent intrinsic apoptosis. Our findings indicated that Chinese herbs will be helpful in promoting the appropriate use of alkylating agent for cancer chemotherapy.

## Supplementary Material

Low, moderate or high dose of HQQD alone could significant reduce the tumor weight as compared with the control mice which suggested an effectiveness of HQQD (Fig S1A), though the inhibition rate was lower than CP administration (Fig S1B). Meanwhile, CP suppressed the thymus (Fig S1C) and spleen index (Fig S1D) which were reversed by individual use of HQQD. These results indicated a protective effect of HQQD alone on CP injury. Data displayed as mean of 10 mice in each group. The experiment was independently repeated for 3 times. ^**^ *p* < 0.01 versus model group, ^***^ *p* < 0.001 versus model group; ^#^ *p* < 0.05 versus CP group, ^##^ *p* < 0.01 versus CP group, ^###^ *p* < 0.001 versus CP group.
