# Supplementary figures and images for: The Hepatoprotective Effect of Haoqin Qingdan Decoction against Liver Injury Induced by a Chemotherapeutic Drug Cyclophosphamide
Source: Evid Based Complement Alternat Med. 2015 May 26;2015:978219. doi: 10.1155/2015/978219 (PMC4460239; doi:10.1155/2015/978219)

**Fig S1**

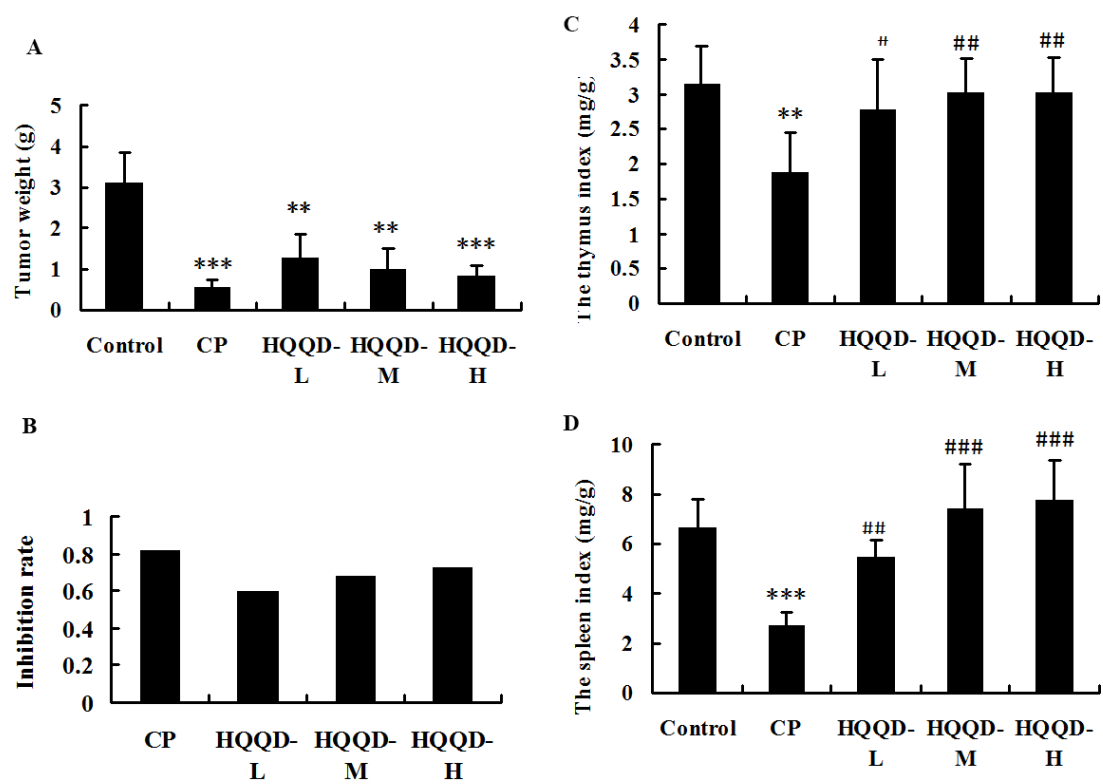

Supplement: Supplementary file 1 — Low, moderate or high dose of HQQD alone could significant reduce the tumor weight as compared with the control mice which suggested an effectiveness of HQQD (Fig S1A), though the inhibition rate was lower than CP administration (Fig S1B). Meanwhile, CP suppressed the thymus (Fig S1C) and spleen index (Fig S1D) which were reversed by individual use of HQQD. These results indicated a protective effect of HQQD alone on CP injury. Data displayed as mean of 10 mice in each group. The experiment was independently repeated for 3 times. ** p < 0.01 versus model group, *** p < 0.001 versus model group; # p < 0.05 versus CP group, ## p < 0.01 versus CP group, ### p < 0.001 versus CP group. [file 978219.f1.pdf]
